# Design paper: The CapOpus trial: A randomized, parallel-group, observer-blinded clinical trial of specialized addiction treatment versus treatment as usual for young patients with cannabis abuse and psychosis

**DOI:** 10.1186/1745-6215-9-42

**Published:** 2008-07-11

**Authors:** Carsten Hjorthøj, Allan Fohlmann, Anne-Mette Larsen, Mette TR Madsen, Lone Vesterager, Christian Gluud, Mikkel C Arendt, Merete Nordentoft

**Affiliations:** 1 Psychiatric Center Bispebjerg, Faculty of Health Sciences, Copenhagen University, Copenhagen, Denmark; 2 Center for Clinical Intervention Research, Copenhagen Trial Unit, Rigshospitalet, Copenhagen University Hospital, Copenhagen, Denmark; 3 Centre for Psychiatric Research, Institute of Clinical Medicine, University of Aarhus, Aarhus, Denmark

## Abstract

**Background:**

A number of studies indicate a link between cannabis-use and psychosis as well as more severe psychosis in those with existing psychotic disorders. There is currently insufficient evidence to decide the optimal way to treat cannabis abuse among patients with psychosis.

**Objectives:**

The major objective for the CapOpus trial is to evaluate the additional effect on cannabis abuse of a specialized addiction treatment program adding group treatment and motivational interviewing to treatment as usual.

**Design:**

The trial is designed as a randomized, parallel-group, observer-blinded clinical trial. Patients are primarily recruited through early-psychosis detection teams, community mental health centers, and assertive community treatment teams. Patients are randomized to one of two treatment arms, both lasting six months: 1) specialized addiction treatment plus treatment as usual or 2) treatment as usual. The specialized addiction treatment is manualized and consists of both individual and group-based motivational interviewing and cognitive behavioral therapy, and incorporates both the family and the case manager of the patient.

The primary outcome measure will be changes in amount of cannabis consumption over time. Other outcome measures will be psychosis symptoms, cognitive functioning, quality of life, social functioning, and cost-benefit analyses.

**Trial registration:**

ClinicalTrials.gov NCT00484302.

## Background

A recent meta-analysis of eleven longitudinal studies concluded that cannabis use is associated with increased risk of lasting psychotic conditions later in life [1]. Assuming causality, as much as 800 annual cases of schizophrenia are preventable in the UK if exposure to cannabis was eliminated [2]. Several studies have shown that use of cannabis increases the risk of developing schizophrenia-like symptoms, especially in young men disposed to developing psychosis [3-10]. Furthermore, use of cannabis among patients with psychosis can maintain and worsen the psychotic symptoms [11-15], and comorbid schizophrenia and substance abuse is associated with lack of compliance to treatment and with more rehospitalizations [15-20]. This indicates both that effective interventions to limit cannabis use in persons with psychosis are needed, and that getting patients to follow such interventions may be difficult.

A randomized trial showed that the combination of cognitive behavioral therapy, motivational interviewing [see [21]], and family involvement had a significant positive effect on level of functioning, psychotic symptoms, and duration of periods of cannabis-abstinence, compared with regular treatment [22]. This finding has been supported by two reviews, concluding that positive evidence exists for integrated treatment with motivational interviews, cognitive behavioral therapy (individual or group-based), and a harm-reduction approach [23,24]. Group-based treatments are arguably less expensive, and in a literature review, Weiss et al. concluded that specialized group therapy could reinforce the effect of an existing treatment [25]. However, there is currently insufficient evidence to show that group-based interventions for cannabis use are superior to individual treatment [26,27]. Similarly, authors of a recent Cochrane review concluded that insufficient evidence exists to show that any psychosocial treatment method for comorbid schizophrenia and substance abuse is superior to others [28].

### Objectives of the CapOpus trial

We plan to undertake a trial in which combined group-based and individual treatment, incorporating motivational interviewing, psycho-education, cognitive behavioral therapy, and social skills training in addition to treatment as usual, is compared with treatment as usual. The primary outcome will be change in number of days abstinent from cannabis within the preceding 30-day period as measured with the Time-Line Follow Back (TLFB) instrument [29,30].

## Methods

**Figure 1:**
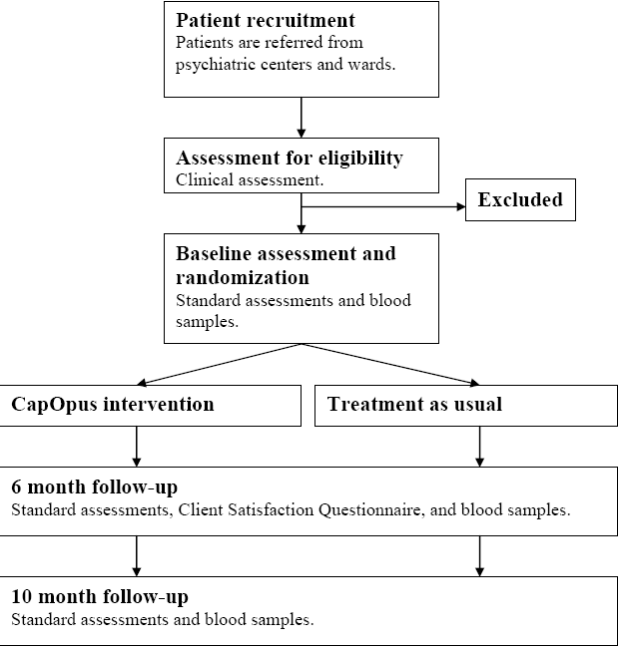
**Flowchart of the CapOpus trial.** See text for further information.

The trial is designed as a two-armed, parallel-group, observer-blinded randomized clinical superiority trial (Figure 1). Patients will receive either specialized CapOpus treatment or treatment as usual, as described below.

### CapOpus intervention – the experimental intervention

**Figure 2:**
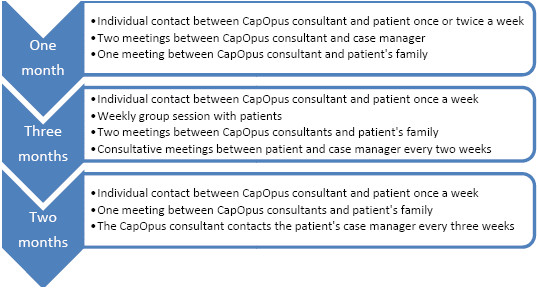
Schematic timeline of the CapOpus intervention.

The contents of the CapOpus intervention are briefly schematized in figure 2. An important part of the CapOpus treatment is to help the patient understand the mechanisms that complicate cessation of cannabis use. The overall target is harm reduction, i.e. that harm from cannabis consumption is thought to be alleviated even if consumption is not terminated, only reduced. This is a method that has proved effective in several studies [23,24]. The CapOpus intervention consists of one month of individual treatment, followed by a three-month group treatment in combination with individual treatment, and followed by two months of individual treatment. The contents of each of these three stages will be described in detail below.

Upon inclusion, the patient's case manager at the referring institution will be offered education and supervision by one of the addiction consultants employed in the CapOpus trial.

During the first month, one of the addiction consultants will be in contact with each patient once or twice a week. A meeting will be held with the patient's family, and there will be contact with the case manager every two weeks. During the three months of group intervention, one of the addiction consultants will have weekly individual contacts with the patient and two meetings with the family. In addition, the patient will follow a weekly group intervention and have consultative contacts with the case manager every two weeks. During the two months following the group intervention, the addiction consultants will be in weekly contact with the patient and the family will be invited to a meeting. The addiction consultants will contact the patient's case manager every three weeks.

For the purpose of enhancing alliance and motivation, the treatment will start with motivational interviewing with the patient being aided in analyzing advantages and drawbacks of continued use of cannabis [21,31]. There is good evidence for the efficacy of motivational interviews in short-term treatment [32,33]. The patient will formulate individual goals for the treatment and be offered the group intervention. The groups will consist of six to eight patients, with the addiction consultants as trainers. Each group will run for three months with weekly sessions lasting 1 1/2 hour. The intervention methods will gradually shift from motivational interviewing to cognitive behavioral therapy as the patients become more motivated to change their cannabis use. There will be emphasis on analyzing advantages and disadvantages of continued use, instructions in coping skills in relation to craving and situations that usually trigger cannabis use, and in developing personal strategies for avoiding or handling these situations.

The group intervention will be followed by two months during which weekly meetings with the patient continue. The addiction consultants offer consultative assistance to the patient's case manager, as well as meeting the patient's family. The purpose is to mediate methods of the intervention to the network surrounding the patient in order to prolong beneficial effects.

To increase adherence to treatment, positive reinforcement will be applied through contingency management in the form of complimentary food and social experiences, which may enhance outcomes of cognitive behavioral therapy or motivational enhancement [34]. Contingency management in the CapOpus trial will have no connection to whether or not the use of cannabis is decreased.

The experimental CapOpus intervention is offered in addition to treatment as usual. The interventions offered to the patients will be registered so that it will be possible to estimate if the two intervention groups receive different forms of treatment as usual.

### Control treatment – treatment as usual

The control treatment consists of receiving the treatment that would have been available without participation in the trial, i.e. treatment as usual. The patient's case manager will carry out the control treatment. In essence, treatment as usual for cannabis abuse is part of the existing, manualized psychosocial and/or pharmaceutical treatment for psychotic illness; however, no explicit manual exists on ways in which cannabis abuse should be approached. Treatment as usual is thus not expected to be uniform. In particular, frequency of contacts may vary between case managers, and this will be registered and analyzed. However, the heterogeneous nature of the control treatment accurately reflects the current situation in Denmark, and we therefore consider this to be the appropriate treatment for analytical control.

Treatment as usual is carried out in one of three established facilities; the OPUS teams for early detection and treatment of young people with psychosis [35], the community mental health centers (CMHC) [36], and the assertive community treatment (ACT) teams [37]. The effect of treatment as usual on cannabis abuse in Denmark is unknown, but a randomized trial comparing OPUS treatment with other treatment (primarily CMHC) investigated prevalence of any comorbid diagnosis of harm or dependence syndrome at baseline and at one and two years follow-up. At baseline, 27% in both treatment arms had such a comorbidity, which decreased to 16 – 17% for OPUS patients and 21 – 22% for other patients [38].

### Recruitment and criteria for inclusion and exclusion

Physicians and therapists in the Danish municipalities of Copenhagen and Frederiksberg (henceforth collectively referred to as Copenhagen) are informed of the CapOpus trial through meetings and leaflets. In particular, OPUS, CMHC, and ACT-teams are targeted. Patients referring themselves and patients referred by general practitioners will be associated with one of these institutions prior to inclusion.

Inclusion criteria are presented in Table 1. The diagnostic criteria are based on the ICD-10 system [39].

**Table 1:** Criteria for inclusion and exclusion in the CapOpus trial.

Inclusion:	Exclusion:
ICD-10 diagnosis F2 (F20–F29): Schizophrenia, schizotypal and delusional disorders	ICD-10 diagnosis F10.2: Alcohol dependence syndrome
	ICD-10 diagnosis F11.2: Opioid dependence syndrome
ICD-10 diagnosis F12: Mental and behavioral disorders due to use of cannabinoids	ICD-10 diagnosis F14.2: Cocaine dependence syndrome
Aged 18–35 years	Refusal to give informed consent
Residence in the Copenhagen area	
Sufficient Danish-speaking capabilities	
Able and willing to give informed consent	

The age group is selected both to ensure a relatively homogenous group of patients, and to reflect the age group at highest risk of developing schizophrenia [40]. The criterion for residence in the Copenhagen area is an attempt to maximize adherence to treatment by minimizing the distance to treatment facilities. If this criterion leads to insufficient numbers of included patients, expansion of the geographic area of the trial will be considered.

Patients must speak and understand Danish sufficiently to participate in both treatment and assessment without an interpreter.

The diagnostic criteria for exclusion are chosen so as to decrease the risk of treatment effects becoming confounded by the presence of other dependence syndromes. Only dependence syndromes of alcohol, opioids, and cocaine are cause for exclusion – more sporadic uses of these substances are not.

### Assessments

**Figure 3:**
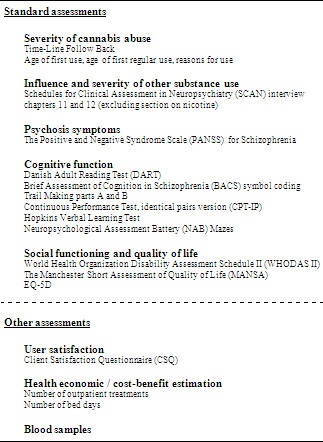
Assessment tools used in the CapOpus trial.

Patients are subjected to three almost identical assessments. The first assessment occurs at baseline before randomization, because information from the baseline assessment is used to perform stratified randomization and to validate inclusion and exclusion criteria. The second assessment occurs six months after the baseline assessment, when the treatment programs have just finished. The final assessment occurs ten months after the baseline assessment, in order to evaluate longer-term effects of the treatments. The contents of the assessments are outlined in figure 3 and explained in the following sections. The standard assessments are conducted at all three assessment points.

#### *Severity of abuse

Assessment of number of days with cannabis use in the past 30 days will be measured at all three assessment points using the TLFB [29]. The TLFB has been reported to have high validity in measuring cannabis consumption when compared with urinalysis [30]. Briefly, the TLFB is a self-reported measurement tool in which the respondent plots all memorable dates within a preceding period on a calendar, and then uses these dates to aid in remembering consumption of a given substance on each day in the period. If the patients consent, self-reported cannabis consumption will be validated with blood samples.

Apart from using the TLFB, information will be obtained about age of first use of cannabis, age of first regular use of cannabis, and reasons for use of cannabis.

#### *Influence and severity of other substance use

The WHO's Schedules for Clinical Assessment in Neuropsychiatry (SCAN) interview's chapters 11 and 12, excluding the section on nicotine, will be used to estimate influence and severity of other substance use, as well as influence of cannabis abuse [41]. Chapter 11 measures influence and severity of alcohol use, and chapter 12 of other psychoactive substances, including prescription medicine.

#### *Psychosis symptoms and clinical improvement

Psychosis symptoms are assessed by the use of The Positive and Negative Syndrome Scale (PANSS) for schizophrenia [42]. Changes in PANSS scores have been shown to correlate well with clinical improvements as measured by the Clinical Global Impressions Ratings (CGI) scale [43-45]. Furthermore, the overall impression of symptoms and functioning is evaluated using the Global Assessment of Functioning (GAF) scale [46,47].

#### *Cognitive function

Various aspects of cognitive function will be measured using a range of psychometric instruments. Prepsychotic intelligence will be estimated using the Danish Adult Reading Test (DART), a Danish adaptation of the National Adult Reading Test [48]. Speed of information processing will be assessed using the Brief Assessment of Cognition in Schizophrenia (BACS) symbol coding and Trail Making Part A [49-51]. Attention and vigilance are assessed by Continuous Performance Test, Identical Pairs version (CPT-IP) [52]. Working memory is assessed using Trail Making Part B. Memory and verbal learning are assessed with Hopkins Verbal Learning Test [53]. Executive functioning is assessed with Neuropsychological Assessment Battery (NAB) Mazes [54].

#### *Social functioning and quality of life

Social functioning within the major life areas of community, social, and civil life will be estimated using the 12-item interviewer administered version of the World Health Organization's Disability Assessment Schedules (WHODAS-II) [55]. Two measures of quality of life (QOL) will be used in a combined attempt to compare QOL improvements in the two treatment arms, namely the five-item EQ-5D [56] and the seventeen-item Manchester Short Assessment of Quality of Life (MANSA) [57].

#### *Sociodemographic variables

A number of sociodemographic variables will be assessed, including age, sex, living conditions, marital status, education, employment status, number of children, ethnicity, and history of homelessness.

#### *Other assessment tools

All of the assessment tools mentioned above will form the standard assessments that will be used at all three assessment points and on all patients. In addition, a few other assessment tools will be used. At six months follow up, user satisfaction with the received treatment will be evaluated using the eight-item Client Satisfaction Questionnaire (CSQ) [58]. Adherence to the CapOpus intervention will be measured by number of contacts between CapOpus consultants on one side and the patient, the patient's family, and the patient's case manager on the other side. Finally, cost of treatment and cost-benefit estimation will be performed by comparing number of outpatient treatments and number of bed days in the two treatment arms. This will be done register-based in The Danish Psychiatric Central Register [59,60] and The Danish National Hospital Register [61], as all persons in Denmark are easily identifiable in registers using a unique personal identification number given to all people either at birth or upon migration into Denmark [62].

### Randomization and blinding

Randomization into one of the two treatment arms is performed after the baseline assessment. Firstly, randomization is stratified by severity of cannabis abuse as measured by TLFB, by creating two sub-strata; one of patients having used cannabis less than 15 days in the preceding 30 day period, and one of patients having used cannabis 15 days or more. This is done to avoid the risk of heavy-users of cannabis being overrepresented in either of the treatment groups. Similarly, randomization will be stratified based on the referrer – OPUS, CMHC, or ACT, as it is unlikely that patients from these three referrers are comparable with regard to psychotic symptoms and level of functioning.

The Copenhagen Trial Unit (CTU) will perform the centralized randomization, and only the CTU will know the block size used to randomize. A research assistant provides information on the patients to the CTU after conducting the baseline assessment. The CTU then performs the randomization procedure based on computer-generated allocation sequences, and communicates the allocated treatment to the CapOpus consultants responsible for the CapOpus experimental intervention. If a patient is allocated to CapOpus intervention, the consultants contact the patient. Regardless of allocation, the consultants also contact the patient's case manager, so that the treatment as usual intervention can be commenced. This procedure ensures that the research assistant, who will be doing the subsequent patient assessments, is blinded to the allocated intervention. Blinding is maintained until the end of the trial. Patients are instructed not to reveal details that may cause the research assistant to deduce which treatment they are receiving. At each of the follow-up assessments, the research assistant will register a guess as to which treatment the patient is receiving. When the trial is finished, inter-rater reliability between the actual treatment and the research assistant's guess will be estimated using Cohen's kappa coefficient [63] to assess the degree to which blinding has been successful.

### Sample size and power calculation

A 2008 Cochrane review of psychosocial interventions for people with both severe mental illness and substance misuse [28] identified five papers with results from studies using a combination of motivational interviewing and cognitive behavioral therapy.[22,64-67] However, only one of these contains information about reduction of days with cannabis abuse, but presents only the median, not the mean, and the range, not the standard deviation.[22] However, 6 months after start of treatment, this particular paper reported that those receiving integrated care had, as median, 20.62 percent of days abstinent from all substances, compared to 1.10 in the routine care group, i.e. a difference of 19.5. Extrapolating this to our study, with a TLFB-period of 30 days, corresponds to 5.85 days. Truncating this to a more conservative 5 days, and assuming a standard deviation also of 5 days, it becomes possible to calculate sample size for an independent samples t-test; with an α-level of 0.05 and a power of 0.90, the required number of patients in each intervention can be calculated to be 22. Due to attrition, more patients will be required. Results from the OPUS trial, which may be comparable to our study in terms of participant profiles, showed that high attrition may be expected among substance-using people with schizophrenia [38]. We obtained data about one-year dropout from the OPUS trial among people with substance misuse, and found that among those receiving the control treatment, the dropout-rate was 37%. Generalizing this to our study, regardless of intervention allocation, at least 35 patients will be required in each of the two intervention groups. However, we aim to include between 60 and 70 patients in each group, as this would enable us to stratify the analyses. With 60 or 70 patients, it should also be possible to measure even smaller differences in secondary outcomes.

### Statistics

The main null-hypothesis to be tested is that there is no difference between the two treatment arms with regard to decrease in cannabis use. All randomized patients will be analyzed, including those who stop receiving treatment, according to the intent-to-treat principle [68]. Continuous outcome measures, including the primary outcome of reduction of days with cannabis abuse, will be analyzed using multivariate analysis of variance (ANOVA) mixed models with repeated measurements. This will include interaction analyses of time and type of treatment, in order to evaluate the effect over time. Binary outcome measures will be analyzed using multivariate logistic regression models. The subgroup of patients who complete the entire experimental treatment will be compared with all those randomized into the trial in order to determine risk factors for attrition. Sensitivity analyses will be carried out to evaluate the effect of treatment in patients that are inaccessible for follow-up. This will be conducted by multiple imputation methodology both using last observation carried forward, and assuming an increase in cannabis use among those lost to follow-up.

### Ethical considerations

The regional ethics committee for the greater Copenhagen area has approved the protocol under the file number H-D-2007-0028, as has The Danish Data Protection Agency under the file number 2007-41-0616. The trial is registered at ClinicalTrials.gov as NCT00484302. Both positive and negative findings from the trial will be published, in accordance with the CONSORT guidelines [69,70].

Information about the trial is presented to potential participants both verbally and in written form. It is stressed that participation is voluntary, without expected negative side effects, and that the patient can withdraw his or her consent at any time without consequence for treatment possibilities. Patients will receive a copy of their rights.

## Discussion

The centralized randomization procedure offers strengths to the CapOpus trial by reducing the risk of selection and allocation bias [71,72]. The outcome assessments are blinded with the intent of being unbiased [71,72]. The planned intense follow up of patients coupled with intention-to-treat analyses should guard against attrition bias [71,72]. Also, the broad array of outcome estimates allows us to measure effects of treatment in many areas of life.

Apart from gathering evidence about best practice in treatment of cannabis abuse among young people with psychosis, the study will allow us to validate the TLFB measurement tool for cannabis against blood samples, something that to our knowledge has not been done previously.

Hopefully, the CapOpus intervention will carry positive effects. If this is so, this could substantially improve treatment of these patients. On the other hand negative consequences cannot be ruled out. The extra interventions in the experimental arm may potentially cause more patients to drop out from usual treatment. Should this occur, the addiction consultants in CapOpus will actively support the patients in resuming contact with the case managers.

Our trial may have several limitations. First, it is a rather small trial aiming at demonstrating a substantial intervention effect. In a sense, our trial may be viewed as a pilot for potentially larger trials at a later stage. Second, the large number of outcomes makes interpretation of significant findings difficult. Accordingly, our interpretation of significant findings at or close to the conventional level of p = 0.05 will be conservative. One further limitation of the trial is that there may be a crossover relating to the patients' case-managers. Due to the randomization procedure, the same case manager may have patients in both the experimental intervention and the control treatment arms. This spawns the risk that elements from the experimental intervention are incorporated into the treatment of patients in the control arm, potentially leading towards type II errors. It will be attempted to bypass this limitation by investigating outcome differences between control-group patients whose case managers also have patients in the CapOpus experimental intervention arm, and those whose case managers do not.

The fact that the current treatment in Denmark is not uniform is reflected in the control treatment, as no attempts will be made to artificially introduce a level of homogeneity in this intervention arm. This may, however, lead to a degree of selection bias if only a small proportion of eligible case managers actually refer patients to the trial. Results will be interpreted accordingly.

## Competing interests

The authors declare that they have no competing interests.

## Authors' contributions

All authors were involved in the conception and design of the study protocol, drafting or revising the manuscript, and have approved the final manuscript. AF, A-ML, and MTRM are responsible for conducting the CapOpus intervention. CH, AF, A-ML, MTRM, and MN are responsible for inclusion of patients. CH is responsible for research interviews and data analysis. MN, MCA, and CG are co-responsible for data analysis. All authors will participate in interpretation of results.
